# Yeast Pre-rRNA Processing and Modification Occur Cotranscriptionally

**DOI:** 10.1016/j.molcel.2010.02.024

**Published:** 2010-03-26

**Authors:** Martin Koš, David Tollervey

**Affiliations:** 1 Wellcome Trust Centre for Cell Biology, University of Edinburgh, Edinburgh EH9 3JR, UK

**Keywords:** RNA, SYSBIO, DNA

## Abstract

To better understand yeast ribosome synthesis, we developed techniques for the rapid harvesting and analysis of metabolically labeled cultures. Modeling of the resulting kinetic data allowed predicted lifetimes and processing patterns to be compared with the experimental data. This supported a transcription time for the 35S primary transcripts of ∼170 s at 30°C (∼40 nt s^−1^), with a high fraction (∼70%) of nascent transcripts cleaved at the early processing sites that generate the 20S precursor to the 18S rRNA. This level of nascent transcript cleavage apparently conflicted with previous reports that modification of yeast pre-rRNA exclusively occurred on released transcripts. A second round of high-resolution kinetic labeling showed that 20S pre-rRNA predominately undergoes methylation as nascent transcripts, whereas the 27S precursor to the 25S/5.8S rRNAs was partially methylated on the nascent transcript. The results demonstrate that quantitative analyses of pre-rRNA processing can yield important biological insights.

## Graphical abstract

**Graphical Abstract:**

**Figure:**
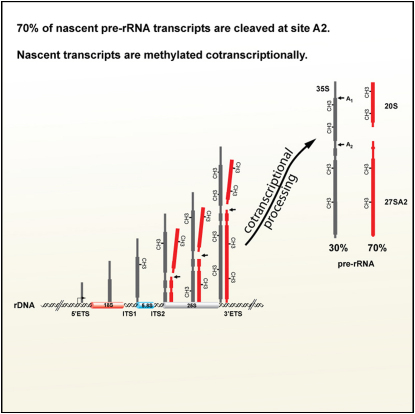

**Highlights:**

► Fast labeling was developed to provide kinetic data for modeling of rRNA synthesis ► The model indicated a high degree of cotranscriptional pre-rRNA cleavage ► This suggested that rRNA modification would also occur on nascent transcripts ► Further labeling confirmed substantial methylation of nascent pre-rRNA transcripts

## Introduction

The synthesis of eukaryotic ribosomes is a remarkably complex system, with multiple intermediate steps in maturation of the rRNAs that involve a vast number of *trans*-acting factors (reviewed in Henras et al., 2008). Moreover, ribosome synthesis consumes a large fraction of the resources in a rapidly growing yeast cell and occupies a pivotal position in cell metabolism (reviewed in Dez and Tollervey, 2004; Warner, 1999). Despite a large research effort over many years there remain many unanswered questions, even concerning basic aspects of the pre-rRNA processing pathway.

Among the earliest studies of yeast ribosome synthesis were quantitative analyses using metabolic labeling and the determination of peaks of radioactivity in preribosomal particles (Trapman and Planta, 1975; Trapman et al., 1975; Udem and Warner, 1972). However, the resolution of different pre-rRNA species on gradients was limited and the analyses difficult. Since the development of gel electrophoresis, analyses of pre-rRNA processing using metabolic labeling have largely been qualitative, with interpretations based on visual inspection of alterations in the intensities of bands displayed on gels. The interpretation of observed changes in pre-rRNA and rRNA levels, for example in mutant yeast strains, was largely intuitive—if a specific mutation leads to accumulation of a particular pre-rRNA processing intermediate, then the defective gene product is assumed to play some role in the onward processing of the accumulated pre-rRNA species. This approach has been applied frequently and with many notable successes. It is, however, quite subjective and complicated by the observation that most mutations have effects on multiple pre-rRNA species, yielding results that can rarely be fully reconciled with any simple model.

With the aim of allowing more rigorous analyses of pre-rRNA processing, we have developed improved quantitative assays and applied mathematical modeling to the data thus obtained. Most modeling techniques require kinetic data as input. For the rRNAs this has long been obtained by metabolic labeling of total RNA in pulse-chase experiments, which works well because the pre-rRNAs represent a large fraction of overall RNA synthesis. There is, however, a major drawback in conventional pulse-labeling techniques as they have been applied over the past 35 years. The problem arises from a discrepancy between the timescale of labeling, harvesting, and RNA extraction, which is in minutes, and the timescale of processing of the many pre-rRNA species, which in several cases is in seconds. In developing quantitative approaches we therefore also sought to shorten the timescale of the analyses to allow sampling on timescales closer to the lifetimes of the processing intermediates. The basis of metabolic labeling is the addition of a radiolabeled compound to mark newly synthesized pre-RNAs in order to follow their subsequent maturation. Most commonly, [5,6-^3^H] uracil is added in order to label the RNA backbone cotranscriptionally, or [methyl-^3^H] methionine is added to label methyl groups that are covalently attached to the pre-rRNA following transcription. The RNAs were then separated on gels, transferred to nylon membranes, and visualized by fluorescence, which was difficult to quantify accurately. However, imaging screens now allow very accurate quantification of tritium incorporation, opening the way for more precise analyses.

Here we hoped that applying the techniques of systems biology would allow us to address several outstanding questions concerning yeast ribosome synthesis: (1) What is the rate of transcription elongation in vivo? (2) What are the life-times of the processing intermediates? (3) To what extent does the 35S pre-rRNA undergo cotranscriptional cleavage of the nascent transcript versus posttranscriptional cleavage of the released transcript? In fact, groundbreaking early studies of yeast ribosome synthesis (Trapman et al., 1975; Udem and Warner, 1972) had apparently answered question three, and it was long believed that cleavage exclusively occurred following release of the completed 35S pre-rRNA. However, EM visualization of “Miller” chromatin spreads indicated that nascent transcript cleavage (NTC) did occur in yeast (Osheim et al., 2004).

The pre-rRNA undergoes many covalent nucleotide modifications, including the addition of 67 methyl groups. Most of these are added to the 2′-hydroxl position on the ribose ring, at sites selected by base pairing to the box C+D class of snoRNAs. The bulk of pre-rRNA methylation was reported to occur in a short period immediately following transcription termination (Trapman et al., 1975; Udem and Warner, 1972), but a small number of base-methylation reactions take place on late, cytoplasmic preribosomes. This conclusion was broadly supported by many published, conventional pulse-chase analyses using [methyl-^3^H] methionine to label newly incorporated methyl groups. However, the substantial level of cotranscriptional processing of the 35S pre-rRNA revealed by our analyses implied that cleavage was either accompanied or preceded by RNA methylation of nascent transcripts. This was addressed and confirmed in a second round of experimental analysis and modeling.

## Results

### Time-Resolved Metabolic Labeling

**Figure 1:**
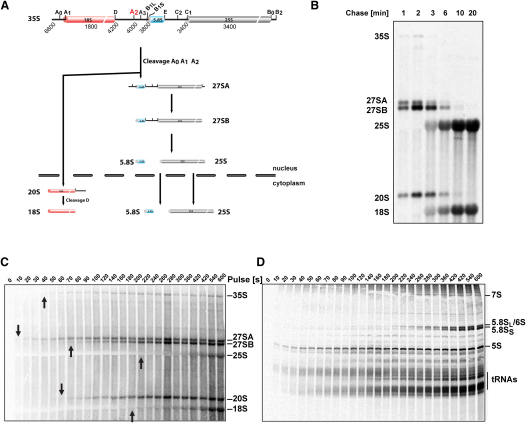
Comparison of Traditional and Fast Metabolic Labeling Techniques (A) Pre-rRNA processing intermediates that are detected by metabolic labeling. (B) Traditional pulse-chase experiment. Cells were pulse-labeled for 1 min with ^3^H uracil and then chased with excess of cold uracil. Total RNA was extracted, separated by gel electrophoresis, transferred to nylon membrane, and exposed to an imaging plate. (C and D) Cells were labeled with ^3^H-uracil and harvested by fast sampling technique. Total RNA was separated by electrophoresis in (C) agarose gel or (D) polyacrylamide gel. The time of initial appearance of individual pre-rRNAs is indicated by arrows. Note that the time of first appearance of 5.8S is ∼200 s. The species migrating at this position in earlier samples is not 5.8S, since it was not coprecipitated with an oligo that depleted mature 5.8S by 90%–98% (data not shown).

Only a subset of pre-rRNA species can be detected following metabolic labeling (shown schematically in Figure 1A). In conventional pulse-chase labeling, cells are pulse-labeled with either [5,6-^3^H] uracil or [methyl-^3^H] methionine, typically for 1 or 2 min, and then chased with a large excess of unlabeled uracil or methionine, with samples taken at various time points. Due to limitations on handling and harvesting of the culture, the earliest chase time that could be routinely analyzed was around 1 min. This is, however, substantially longer than the lifetime of short-lived pre-rRNAs including the 35S pre-rRNA. In consequence, several pre-rRNA species appeared simultaneously at the earliest time point (Figure 1B). This greatly limited the utility of metabolic labeling for quantitative kinetic analyses of pre-rRNA processing.

We therefore sought to develop techniques that would allow analyses of metabolic labeling on much shorter timescales. Our protocol (see the Experimental Procedures) involves the transfer of samples taken from the labeled culture directly into ethanol at −80°C. This approach allowed us to routinely harvest samples at 10 s intervals, and shorter time points would be feasible if necessary. To facilitate modeling of the data, we analyzed only the incorporation of label, without the addition of an unlabeled chase (Figures 1C and 1D). In these analyses we therefore assess the approach to steady state, a long-established methodology for determining the lifetimes of transient intermediates.

Using this labeling technique, we could readily resolve the time of initial appearance of all detectable pre-rRNA intermediates (arrows in Figure 1C). The 27SA pre-rRNA appeared first (10–20 s), then 35S (40 s), 20S (50 s), and 27SB (60–70 s). Inspection of these data immediately revealed an unexpected result; the order of appearance of labeled pre-rRNAs does not reflect their order of formation in the ribosome synthesis pathway (Figure 1A). Labeling the cells with [^3^H] adenine gave the same pattern, showing that this is not specific to uracil labeling (see Figure S1 available online).

**Figure 2:**
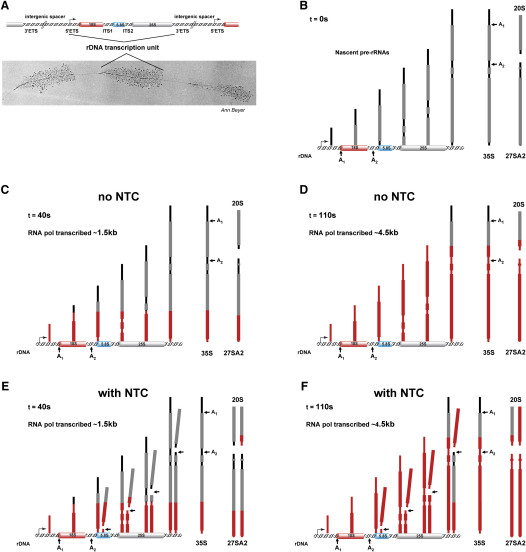
Diagram Showing the Progression of Pre-rRNA Labeling with and without Cleavage of the Nascent Transcript (A) A Miller Christmas tree allows the visualization of the nascent pre-rRNAs generated as multiple RNA Pol I molecules move along the rDNA. (B) At the time of label addition T_0_, the nascent pre-rRNAs are entirely unlabeled. (C) After 40 s of labeling, each transcribing Pol I has moved ∼1.5 Kb. The 18S and 20S regions of the pre-rRNA have been transcribed, but in the absence of NTC, these labeled sequences are all within nascent transcripts and are not yet detectable as discrete RNA species. (D) After 110 s of labeling, each transcribing Pol I has moved ∼4.5 Kb. Labeled 20S generated by RTC is now detected. (E) After 40 s of labeling with NTC, labeled 20S pre-rRNA is released from the nascent transcript and is detected ∼90 s before 20S that is generated by RTC. (F) After 110 s of labeling with NTC, labeled 20S is generated by both NTC and RTC, and its rate of accumulation is therefore expected to increase.

To understand what was happening during the labeling experiments, it was necessary to reconsider how individual pre-rRNAs are labeled and released. As can be visualized in Miller spreads of yeast rDNA (Figure 2A; image kindly provided by Ann Beyer, University of Virginia), each rDNA carries multiple Pol I molecules (Osheim et al., 2009). Following addition of [^3^H] uracil (at T_0_), each nascent pre-rRNA is progressively labeled toward the 3′ end—starting from the position occupied by the transcribing polymerase at T_0_. The lifetime of the fully transcribed 35S was predicted to be very short (Venema and Tollervey, 1999), and this was confirmed by our analyses (see below), so the entire 35S pre-rRNA population should be quite homogeneous with respect to labeling at any given time point. Figures 2B–2F show the predicted differences in the timing of the appearance of labeled 20S pre-rRNA in the presence and absence of cotranscriptional cleavage of the nascent transcript. The 20S pre-rRNA is generated by cleavage of the 35S pre-rRNA at sites A_1_ and A_2_. If cleavage occurs only posttranscriptionally on the released 35S transcript (*r*eleased *t*ranscript *c*leavage, RTC), labeled 20S should be first detected only ∼100 s after label equilibration (using our calculated transcription rate of ∼40 nt s^−1^; see below). This is the time needed for the transcribing polymerase to traverse the region from A_2_ to the 3′ end of the 25S rRNA where transcript release occurs. During this period, only nascent pre-rRNA transcripts are labeled over the 20S region. In contrast, 20S generated by cotranscriptional NTC will be detected at earlier time points, with the exact timing depending on the delay between the transcription of site A_2_ and its cleavage. Inspection of the primary data (Figure 1C) shows that 20S is detected at ∼60 s. This is too early to be derived only from posttranscriptional cleavage of the 35S pre-rRNA and must be a result of NTC.

The existence of NTC can therefore be demonstrated by logical deduction following simple inspection of the kinetic data.

### Quantification and Modeling of Metabolic Labeling Data

**Figure 3:**
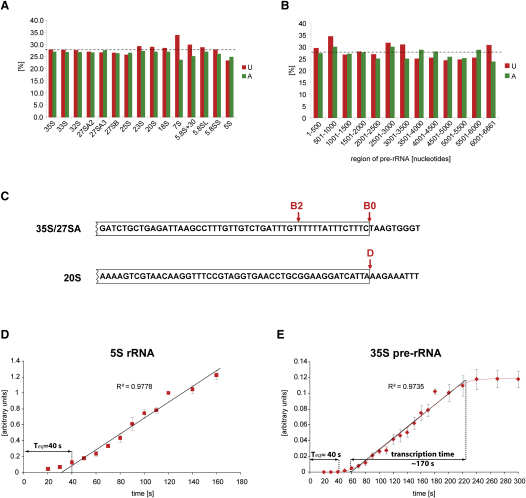
Determination of the Time Required for Label Equilibration (A) Distribution of uridine and adenosine in pre-rRNAs. (B) Distribution of uridine and adenosine in 500 nt segments of primary pre-rRNA transcript. (C) Sequence comparison of the ends of 35S/27SA and 20S pre-rRNAs. (D) Labeling of the 5S rRNA. Estimated time of intracellular uracil pool is shown. Linear regression trend line is represented by a dashed line. Mean of two experiments. Error bars represent standard error. (E) Labeling of the 35S pre-RNA. Estimated time of transcription of 35S is indicated. Linear regression trend line is represented by a dashed line. Mean of four experiments. Error bars represent standard error.

To better determine what fraction of the nascent transcripts undergoes cleavage, the data were quantified and modeled. When formulating the equations, we assumed that the intensity of each pre-rRNA is directly proportional to the length of the region transcribed and therefore labeled by incorporation of radioactive uridine. Figures 3A–3C show that, with the exception of the 3′ end of 35S/27SA precursors, the distribution of uridine or adenine in various pre-rRNAs is sufficiently even to allow this simplification. We also assumed that the speed of transcription is constant across the whole rDNA unit. This assumption is probably not entirely correct, however; any major deviations from the assumed constant speed would show up as discrepancies between the model and measured data. We also assigned a lifetime to each pre-rRNA species, rather than assuming exponential decay. Early labeling data indicated that each pre-rRNA species has a lifetime (Trapman et al., 1975; Udem and Warner, 1972), and this has been seen in many subsequent analyses. This presumably arises because each visible processing reaction requires a large number of unobserved assembly and reorganization events. These individual steps may themselves have first-order kinetics, but we think that even this is unlikely and have argued for kinetic proofreading activities (Houseley and Tollervey, 2009). These both slow the forward reactions (since the proofreading works partly on the use of a delay to allow noncognate interactions to preferentially dissociate) and leads to preferential degradation of any slowly maturing complexes, sharpening the time profile. In practice, there will presumably be a range of lifetimes for individual molecules, but our data average these over many cells and transcription units.

To model the labeling, we derived a set of simple equations for each RNA species based on transcription rate and time. The complete model runs in MS Excel and can be downloaded from the Supplemental Information in a fully executable and modifiable form. This model allows pre-rRNA transcription and processing to be simulated in the presence or absence of NTC and estimates the percentage of nascent transcripts cleaved. Taking into account the above assumptions, we can write

$$I_{35S}^{t}=\nu t\times \left(1-\text{P}\right),$$

where ***I*** is the amount of radioactivity incorporated into the “just-finished” 35S transcript within the time interval [t − 1,t], ***v*** is speed of transcription in nt.s^−1^, and ***t*** is time. The variable ***P*** is the probability of NTC—if NTC occurs, no 35S is formed. Each completed 35S molecule will be labeled from its 3′ end along a region that is v^∗^t in length (see the scheme in Figure 2). When sufficient time has elapsed for polymerases to transcribe the whole 35S region (6600 nt), all further 35S molecules will be fully labeled and the maximum intensity will have been reached. The following conditions are therefore added:

$$\left(1\right)\;\text{For}\;\text{v}t\;<\;0\;I_{35S}^{t}\;=\;0$$

$$\left(2\right)\;\text{For}\;0\;<\;\text{v}t\;<\;6600\;I_{35S}^{t}\;=\;\nu t\;\times \;\left(1-\text{P}\right)$$

$$\left(3\right)\;\text{For}\;\text{v}t\;>\;6600\;I_{35S}^{t}\;=\;6600\;\times \;\left(1-\text{P}\right)\text{.}$$

At any given time ***t*** after the labeling commences, the total intensity ***I_35S_*** is the sum of all radioactivity incorporated into transcribed molecules within the time interval [0,t]. However, as each molecule of 35S has a mean life-time **τ_35S_** after which it will undergo further processing, we need to sum only molecules “younger” than **τ_35S_**. All previously transcribed molecules were already processed into further intermediates. Therefore,

$$I_{35S}\;=\;\sum_{0}^{t}\;I_{35S}^{t}\;-\;\sum_{0}^{t\;-\;\tau_{35}}I_{35S}^{t}\;=\;\sum_{t-\tau_{35}}^{t}I_{35S}^{t},$$

where $I_{35S}^{t}$ takes one of the three forms described above.

Similar equations can be written for 27S pre-rRNA and 25S rRNA (see Table S1). The equations for 20S pre-rRNA and 18S rRNAs are more complex:

$$I_{20S}\;=\;\sum_{t-\tau_{20S}}^{t}I_{20S\text{\_}RTC}^{t}+\sum_{t-\tau_{20S}}^{t}I_{20S\text{\_}NTC}^{t}\text{.}$$

The model allows for 20S to be produced either by posttranscriptional RTC, where

$$I_{20S\text{\_}RTC}^{t}=\left[\nu \left(t-\tau_{35S}\right)-4000\right]\times \left(1-\text{P}\right),$$

or cotranscriptional NTC following transcription through site A_2_, the 3′ end of 20S,

$$I_{20S\text{\_}NTC}^{t}=\nu \left(t-\frac{L}{\nu }\right)\times \text{P}\text{.}$$

The parameter ***L*** is a distance from the A_2_ site at which the cleavage occurs. We predict that NTC occurs within a region of certain length, “a cleavage window,” downstream of the A_2_ site. The distance L approximates the middle position of such a cleavage window. Note that the pre-rRNA is always cleaved at site A_2_; however, the timing of this cleavage is delayed relative to transcription through site A_2_.

The 20S pre-rRNA is approximately 2000 nt in length and corresponds to the 5′ region of the primary transcript (less the 700 nt 5′ETS), and site A_2_ lies some ∼4000 nt from the 3′ end of the 35S transcript. If <4000 nt have been transcribed following label addition, then no released 35S molecules are labeled over the 20S region and only 20S produced by NTC can be seen. When transcription has proceeded >4000 but <6000 nt, RTC 20S is partially labeled. Only after transcription of >6000 nt is the RTC 20S fully labeled.

Therefore,

$$\left(1\right)\;\text{for}\;0\;<\;\nu \left(t-\tau_{35S}\right)\;<\;4000\;\text{and}$$

$$\left(\text{a}\right)\;0\;<\;\nu \left(t-\frac{L}{\nu }\right)\;<\;2000\Rightarrow I_{20S\text{\_}RTC}^{t}=0\;\text{and}\;I_{20S\text{\_}NTC}^{t}=\nu \left(t-\frac{L}{\nu }\right)\times \text{P}$$

$$\left(\text{b}\right)\;\nu \left(t-\frac{L}{\nu }\right)\;\geq \;2000\Rightarrow I_{20S\text{\_}RTC}^{t}\;=\;0\;\text{and}\;I_{20S\text{\_}NTC}^{t}\;=\;2000\;\times \;\text{P},$$

$$\left(2\right)\;4000\;<\;\nu \left(t-\tau_{35S}\right)\;<\;6000\Rightarrow I_{20S\text{\_}RTC}^{t}\;=\;\left[\nu \left(t\;-\;\tau_{35S}\right)\;-\;4000\right]\times \left(1-\text{P}\right)\;\text{and}\;I_{20S\text{\_}NTC}^{t}\;=\;2000\;\times \;\text{P},$$

$$\left(3\right)\;\nu \left(t\;-\;\tau_{35S}\right)\;>\;6000\Rightarrow I_{20S\text{\_}RTC}^{t}\;=\;2000\;\times \;\left(1\;-\;P\right),\;\text{and}\;I_{20S\text{\_}NTC}^{t}\;=\;2000\;\times \;\text{P}\text{.}$$

All equations are summarized in Table S1.

### Fitting the Model to the Data

Initial analyses with [^3^H] uracil showed fluctuations in labeling of all pre-rRNAs that we attributed to the effects of uracil addition on the uptake activities of uracil permeases, which are known to be subject to complex regulation by multiple mechanisms (Lacroute, 1968; Seron et al., 1999). The labeled uracil represents a final concentration of 1 μM, and it was found that addition of unlabeled uracil to 1 μM 15 min prior to addition of labeled uracil generated more consistent incorporation (data not shown). This pre-incubation did, however, reduce the incorporation of [^3^H] uracil and delayed detection of labeled RNA species by 10–20 s relative to the data shown in Figures 1A and 1B, due to the reduced sensitivity of detection with lower labeling efficiency.

In generating labeling curves, an important parameter is the time required for entry of the labeled uracil into the cell and equilibration of the intracellular pool. To determine this, we examined the labeling of the 5S rRNA, which has a short transcription time (∼2 s) and minimal processing and is stable once synthesized. Labeled 5S was visible after 10 s, and accumulation was linear after ∼40 s (Figure 3D). In the model we used 40 s equilibration time, which corresponds to the time from which a theoretical linear incorporation of label into 5S rRNA would have started (Figure 3D). In principal, labeled 35S should start to accumulate at the same time as 5S rRNA; however, we consistently detected 35S only after 40 s (Figures 1C and 3E). The difference presumably reflects the sensitivity of detection of the two species. The short lifetime of 35S ensures that it does not accumulate and generates a low signal even in rapid labeling experiments. Consistent with this, 27SA was detectable ∼30 s before 35S, whereas the 27SA molecules resulting from NTC at A_2_ should appear simultaneously with 35S, since both are generated by cleavage at site B_0_, and 27SA generated by RTC should appear after 35S. However, the longer lifetime that we predict for 27SA (see below) greatly facilitates its detection. This observation led us to add a “sensitivity constant” into the model, which corresponds to a minimal region of pre-rRNA that needs to be labeled to be detectable. In the pulse labeling, 1 μM ^3^H-uracil is added to the cells. The estimated intracellular pool of uracil is in the 10^−4^ M range (Jund et al., 1977). Therefore, we can estimate that very approximately 1% of uracil incorporated into RNA is radioactive. The distribution of U in the rRNA is even (Figures 3A and 3B), thus 400 nt long transcript will on average carry one radioactive uridine. This minimal region of 400 nt, corresponding to 10 s transcription time, is subtracted from the sum of transcribed regions in all equations.

A complication in quantification of the data arose from the observation that the mature 25S rRNA and, to a much lesser extent, the 18S rRNA quench the ^3^H signal in these regions of the gel. This presumably arises from the high concentration of mature rRNA and the low penetrance of ^3^H-derived beta particles. The effects of quenching could be observed by comparison of the 25S rRNA signals detected using the same amount of radiolabeled RNA mixed with increasing quantities of unlabeled RNA (Figure S2). No quenching was detected for any pre-rRNA species. The degree of quenching could be accurately determined by comparison of the relative signals obtained for RNAs labeled in parallel with [^3^H] and with [^32^P], which is not subject to quenching (see the Supplemental Experimental Procedures). At the RNA concentration used in our analyses (∼2 μg RNA per lane), the 25S rRNA signal was quenched 2.3-fold, and 18S rRNA was quenched 1.2-fold. These correction factors were applied to the labeling values obtained. Since the same amount of total RNA is present in each sample, regardless of the degree of labeling, the same quenching factor can be applied to each sample.

The model contains 14 variables, and many of these are interconnected. It therefore proved impossible to automatically derive numerical values that could be demonstrated to be the best possible fit, either using the Excel-based model or the equivalent model converted to differential equations and executed in the Mathematica program (K. Axt, M.K., and D.T., unpublished data), without utilizing prior knowledge. We therefore initially populated the model with values for the pre-rRNA lifetimes that were previously estimated, by ourselves and others, from the steady-state levels of the pre-rRNAs in northern hybridization relative to the mature rRNAs, which are assumed to have effective life-times close to the doubling time of the yeast. For example, the 35S pre-rRNA was reported to have a lifetime of around 10 s (Venema and Tollervey, 1999), in good agreement with the optimized parameters derived from the model. Similar calculations indicated average overall life-times of 27SA, 27SB, and 20S of approximately 1, 3, and 2 min, respectively. These figures were necessarily quite inexact but are consistent with many published conventional pulse-chase analyses of the type shown in Figure 1B. Discrepancies between the curves generated by the initial values and the experimental data indicated gaps in our understanding of the pathway.

**Figure 4:**
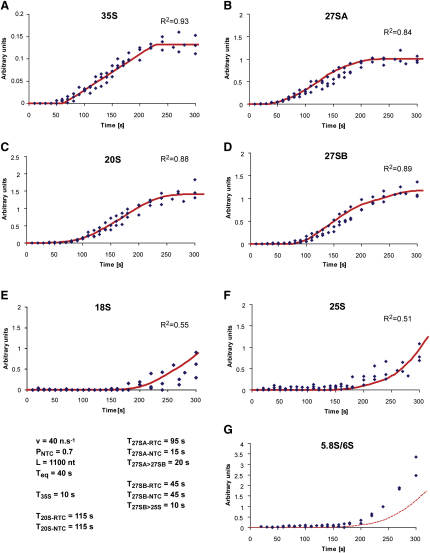
Fitting Model Curves to Experimental Data Diamonds represent experimental data points from three independent experiments. Red curves are predictions of the model. All data and model were normalized to 27SA at 270 s and corrected for recovery judged by levels of 18S and 25S rRNA determined by northern hybridization of the same filter with ^32^P-labeled probes. The dashed red curve in (G) shows the curve for 25S for comparison, since 5.8S is not currently incorporated into the model. Parameters used are shown: v, transcription velocity; P_NTC_, probability of NTC occurring; L, distance from site A_2_ traveled by the transcribing polymerase prior to NTC; T_eq_, time for equilibration of the labeled uracil; T_35S_ etc., the life-times determined for the various pre-rRNA species by fitting of the model to the data. Above each curve a coefficient of determination R^2^ for the predicted curve is shown.

Figure 4 shows the final fit of full model to the various pre-rRNAs and rRNAs obtained by manual adjustment of the initial parameters. Values of all parameters are listed in Table 1. The total times given for synthesis of the rRNAs include the transcription times: 170 s for RTC and 100 s for the NTC 20S (the time for transcription to the region of cleavage at A2 + 1100). These values are also used as the default settings in the model available in the Supplemental Experimental Procedures. The Excel Solver program confirmed that this corresponds to the local minimum, indicating that this is indeed a good fit to the data.

**Figure 5:**
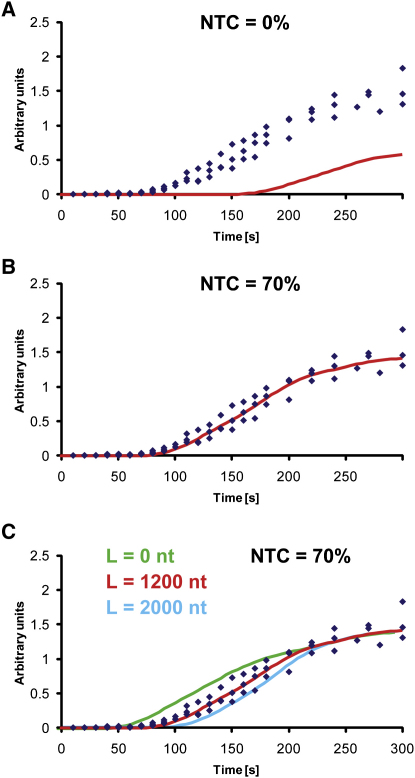
Fitting the 20S Curves with and without Nascent Transcript Cleavage Diamonds represent experimental data points from three independent experiments; curves are predictions of the model. (A) Model prediction for labeling of 20S pre-rRNA without NTC. (B) Model prediction for labeling of 20S pre-rRNA with 70% NTC. (C) Effect of the cleavage window position on 20S curve prediction.

Using the data in Figures 3E and 4A, the in vivo transcription rate of RNA Pol I can be determined robustly from the time required for 35S pre-rRNA labeling to reach steady state. This time was determined to be ∼170 s, corresponding to ∼40 nt s^−1^. 20S pre-rRNA showed a more complex labeling pattern than 35S. The early initial appearance of labeled 20S demonstrates cleavage of the nascent transcript shortly after transcription of its 3′ end, the A_2_ processing site. An inflection in the curve is predicted to correspond to the first appearance of 20S derived from cleavage of the released 35S pre-rRNA, which increases the rate of synthesis of labeled 20S. Altering the values for the efficiency and timing of NTC gives strikingly different predicted curves for the rate of 20S accumulation (Figure 5). A good fit to the experimental data was achieved by setting frequency of NTC to 70%, with cleavage occurring when the transcribing polymerase was 1100 nt downstream of site A_2_. Notably, both the efficiency and timing would be in close agreement with conclusions drawn from EM analyses (Osheim et al., 2004). Cleavage at this location would also explain the ∼30 s delay in the appearance of 20S after 27SA. Due to the 40 s equilibration time for the intracellular uracil pool, we are currently unable to accurately determine the length of the cleavage window. For all 20S species we determined a lifetime of 115 s.

We were unable to find any plausible single lifetime for the 27SA pre-rRNA that would allow a close match between the predicted and experimental data. However, a good fit could be obtained by attributing different lifetimes for the 27SA molecules produced by NTC and RTC. The best fit was obtained with a lifetime of 15 s for the NTC-derived species and 90 s for the RTC-derived species. Our interpretation of these results is that most preribosomes are competent for NTC and rapid subsequent processing. In contrast, a subset of ∼30% follow a different, perhaps slower, assembly pathway that results in RTC and slowed subsequent maturation. With these values for 27SA lifetimes, a lifetime of 45 s was determined for the entire 27SB pre-rRNA population.

### Analysis of Pre-rRNA Methylation

From the model for pre-rRNA processing, we estimated that 70% of the 20S pre-rRNA is generated by NTC. This finding was difficult to reconcile with previously reported findings on the timing of pre-rRNA methylation. Initial analyses reported that methylation, like cleavage, was posttranscriptional but occurred rapidly following release of the full-length 35S pre-rRNA (Trapman et al., 1975; Udem and Warner, 1972). Many subsequent studies have made use of labeling with [methyl-^3^H] methionine (the methyl donor) to follow rRNA maturation. Their interpretation assumed that newly released 35S was rapidly modified, predominately at positions selected by the 73 modification guide snoRNAs, and then cleaved at sites A_1_ and A_2_, the exception being the late, cytoplasmic m_2_^6^A dimethylation of 20S pre-rRNA by Dim1 (Brand et al., 1977; Lafontaine et al., 1994; Udem and Warner, 1973).

**Figure 6:**
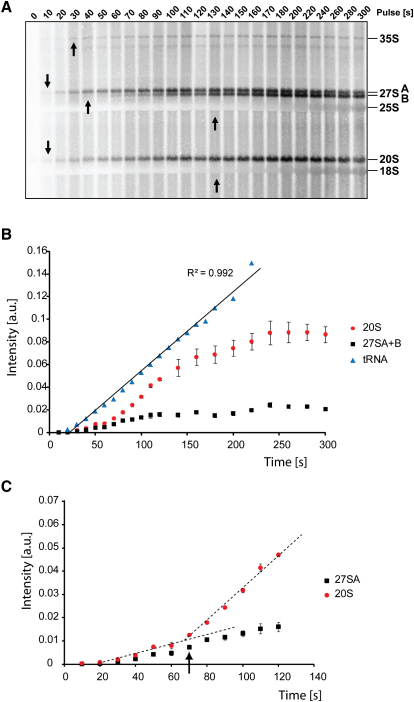
Comparison of tRNA and Pre-rRNA Methylation (A) Cells were metabolically labeled with [^3^H-methyl]-methionine and harvested at times indicated by fast sampling technique. Appearances of individual pre-rRNAs are indicated by arrows. (B) Graph of labeling intensity of tRNAs and 20S and 27SA pre-rRNAs. (C) Detail graph of the first 2 min of labeling. Dashed lines show linear regression trend lines. Arrow indicates position of an inflexion in 20S curve. A mean of three experiments is shown; error bars represent a standard error.

There was, however, a clear problem with this interpretation if 20S is predominately generated by NTC that occurs some 80 s before completion of 35S transcription. One possibility would be that the nascent pre-rRNA was predetermined to have a fate of NTC or RTC, such that only those molecules destined for NTC would bind the modification guide snoRNAs and undergo early modification. However, we considered it more likely that all pre-rRNA transcripts in fact undergo cotranscriptional modification. This cannot readily be addressed for pseudouridine formation, but methylation can be followed by metabolic labeling with [methyl-^3^H] methionine. We therefore applied the fast labeling technique to methionine labeling (Figure 6).

The tRNAs undergo methylation of the released transcript at many positions, and label incorporation into tRNA was strikingly linear following an initial equilibration period of 20 s (Figures 6A and 6B). If pre-rRNA methylation similarly occurs on the released transcript, we should see the same equilibration time followed by linear incorporation, with a plateau at a time point corresponding to the lifetime of the pre-rRNA species. In contrast, if the nascent pre-rRNAs are methylated, we expect a delay in the initial accumulation of labeled RNA, since the newly released pre-rRNA population will already be modified with nonlabeled, snoRNA-directed 2′-O-methyl groups. We also expect an inflection in the 20S labeling curve; initial labeling will represent late, released transcript methylation of previously synthesized preribosomes, with nascent transcript methylation becoming visible after a lag corresponding to the time between modification and transcript release.

In the labeling curves for 20S pre-rRNA, the initial 20 s lag was followed by a low level of incorporation, representing late, cytoplasmic methylation (Figure 6B). A clear inflexion point was observed following labeling for 60 s, demonstrating predominant methylation of nascent transcripts. The inflection is seen ∼40 s after label equilibration. This delay would correspond to transcription of approximately 1.6 Kb prior to the appearance of labeled 20S. This is in fair agreement with the predicted distance from the last methylated nucleotide of 18S to the predicted position of the polymerase at the time of NTC (∼1.4 Kb) derived from the model.

**Figure 7:**
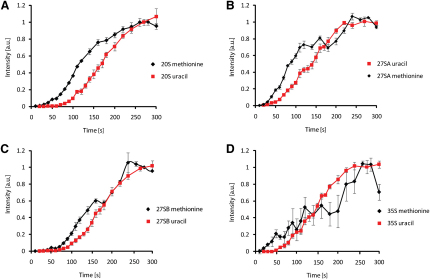
Comparison of Pre-rRNA Labeling with Methionine and Uracil Mean values from three independent experiments for both [5,6-^3^H]-uracil and [^3^H-methyl]-methionine labeling were plotted against time. Data for each precursor were normalized to the value of the final steady state to allow direct comparison. (A) 20S, (B) 27SA, (C) 27SB, and (D), 35S pre-rRNAs. Error bars represent standard errors.

Labeling of the 27SA pre-rRNA was notably different (Figures 6B and 6C). Incorporation was initially linear with a delay of ∼20 s, very similar to that of tRNA, clearly indicating substantial labeling of released transcripts. After 110 s (∼90 s of labeling), a plateau was reached. At this time the entire 27SA population that undergoes released transcript methylation has been labeled, so this represents the life-time of these 27SA species. However, following a lag of ∼70 s, incorporation reproducibly rose again, and this must represent a pool of nascent transcript-methylated 27SA pre-rRNA species. Comparison of uracil and methionine labeling shows the difference between 20S and 27SA labeling (Figures 7A and 7B). The curves for 20S labeling are similar with both labels, demonstrating predominant labeling of nascent transcripts. The difference in timing reflects both the slower equilibration of uracil and the delay between transcription of a sequence and its recognition and methylation by the snoRNPs. In contrast, the discrepancy seen for 27SA labeling shows a substantial degree of released transcript modification. It is notable that the life-time of 27SA population that is methylated on the released transcripts (∼90 s) is in close agreement with the predicted life-time for the 27SA population that is processed from released transcripts.

Similarly to 27SA, both 27SB and 35S pre-rRNAs show an intermediate plateau (Figures 7C and 7D), confirming that these are methylated both as nascent and released transcripts. Note also that the time required to reach steady state in the methionine labeling experiment is very significantly longer than their life-times, particularly for 35S, further demonstrating methylation of the nascent transcripts.

We conclude that the 20S pre-rRNA predominately undergoes methylation of the nascent transcript, while the other pre-rRNAs are partially modified on the nascent transcripts.

## Discussion

Here we report the development of techniques for sampling metabolically labeled yeast cells at 10 s intervals, and the use of the derived quantitative data to populate a mathematical model of the processing pathway.

Perhaps most surprising was the realization that we, and others in the field, had never correctly understood the time course of the appearance of labeled pre-rRNAs during pulse-labeling experiments, despite the publication of many papers that made use of the technique. Since pre-rRNAs are being synthesized by many polymerases, located at all possible positions along the pre-rRNA, we had implicitly assumed that pre-rRNAs partially labeled at all positions would be synthesized at early time points during labeling. In fact, as diagrammed in Figure 2, each pre-rRNA molecule must be transcribed to completion, or until NTC, before it becomes visible as a discrete species in pulse-labeling experiments. At early time points all pre-rRNAs are indeed incompletely labeled—but this takes the form of a 5′ domain of unlabeled RNA and a fully labeled 3′ domain. This realization allowed a more detailed interpretation of pulse-labeling data than previously obtained.

With this insight, the transcription time of 35S synthesis can be reliably determined—from the time required to reach steady state, less the label equilibration time and the 35S life-time, which we knew to be very short (∼10 s) (Venema and Tollervey, 1999). At 30°C in our strain (which is derived from W303), this was found to be 170 s, corresponding to 40 nt s^−1^. The in vivo transcription rate for RNA Pol I was previously estimated at 60 nt s^−1^, based indirectly on the overall rate of ribosome synthesis and the number of transcribing polymerases (French et al., 2003).

To better understand the data, we produced a mathematical model. The aim of the model was to capture our current understanding of the processing pathway and allow this to be tested against the experimental data. Manually fitting the model to the experimental data indicated that 70% of pre-rRNAs undergo NTC (Table 1). The cleavage that releases the pre-rRNA occurs at site A_2_, but a large number of analyses show that this is normally preceded by cleavage at sites A_0_ and A_1_, and we assume that these also occur on the nascent transcript. At first sight it seemed surprising that such a high level of cleavage had not been detected in previous biochemical analyses of pre-rRNA processing. However, a striking feature of the data is the minimal effects of altering the fraction of NTC pre-rRNA on the predicted curve of 35S labeling. The major difference in going from 0% to 70% NTC is that the predicted lifetime for 35S pre-rRNA changes from 3 to 10 s. Processing on this timescale was not accessible to any previous kinetic analysis. The data clearly indicate that the nascent transcripts are cleaved at site A_2_ after the polymerase has transcribed a further ∼1 kb. These data are in strikingly close agreement with conclusions drawn from analyses of chromatin spreads (Osheim et al., 2004). These estimated that up to 79% of pre-rRNAs undergo NTC in early log phase cells (the conditions used here), with cleavage generally occurring when the transcribing polymerase was traversing the 5′ region of the 25S rRNA gene.

An obvious question is why cotranscriptional cleavage was not observed in the heroic early analyses of ribosome synthesis, which concluded that cleavage was entirely posttranscriptional (Trapman et al., 1975; Udem and Warner, 1972). Looking at the methodology the clearest difference is that, whereas the analyses reported here were performed using unperturbed, exponentially growing cells, the original analyses were performed using cells that had been spheroplasted (i.e., their cell walls had been removed by snail gut extract) and were growing in medium containing 400 mM MgSO_4_ as osmotic support. Moreover, in experiments to show precursor product relationships between the different pre-rRNA species, the cells were additionally subjected to heat shock to delay processing. It seems possible that these treatments also inhibited NTC.

Cleavage at site A_2_ also generates the 27SA pre-rRNA, which is then processed to 27SB pre-rRNA and the 5.8S and 25S rRNAs. We were unable to find any feasible lifetime for the 27SA that would allow the predicted accumulation of 27SA or 27SB to accurately fit the experimental data. A good match for both species could, however, be obtained by assuming the existence of two populations of 27SA with different lifetimes. The best fit was obtained by setting the lifetime of 30% of the 27SA population at 90 s, while the remainder has a lifetime of 15 s (Table 1). In the case of 27SA, unlike 20S, the kinetics of appearance of the pre-rRNAs derived from NTC and RTC are identical, so we cannot distinguish between these species in our analyses. However, we speculate that the 30% with slow processing kinetics represent the RTC-derived 27SA population. The features of the pre-60S particles that prevented NTC may also delay subsequent processing after RTC.

The subsequent maturation of 27SB to the 5.8S and 25S rRNAs could be fitted by assuming a single lifetime for the entire 27SB population. However, there appears to be a significant delay between the loss of 27SB and the appearance of the mature 25S rRNA. We are unable to detect or resolve the intermediate 26S and 25S′ pre-rRNA species in our current analyses, so different approaches will be needed for their incorporation into the model.

The conclusion that substantial NTC takes place was difficult to reconcile with previous reports that pre-rRNA modification was entirely posttranscriptional. We therefore investigated the kinetics of rRNA modification using [methyl-^3^H] methionine labeling, which is the initial methyl donor for all methylation reactions in the cell. The kinetics of pre-rRNA and rRNA methylation was compared to tRNA methylation, which occurs entirely on the released transcript, and to uracil labeling, which is entirely cotranscriptional. This revealed that methylation over the 18S rRNA (shown by labeling of the 20S pre-rRNA) predominately takes place on the nascent transcript. A low level of released transcript methylation was observed, presumably corresponding to the late cytoplasmic dimethylation of the 3′ end of 18S by Dim1 (Brand et al., 1977; Lafontaine et al., 1994; Udem and Warner, 1973).

Methylation over the 25S rRNA, revealed by labeling of the 27SA pre-rRNA, showed a more complex pattern. The rapid initial labeling revealed a pre-rRNA population that is methylated on released transcripts and has a life-time of ∼90 s. Subsequent delayed labeling reflects an additional, nascent transcript-methylated population. It is notable that modeling suggested that 27SA pre-rRNA generated by RTC also has a life-time of ∼90 s. While we are unable to demonstrate that these represent the same populations, the data fit well with the model that 27SA generated by RTC undergoes both slow maturation and posttranscriptional methylation. Notably, labeling experiments indicated that nascent transcript methylation also takes place in human cells (Greenberg and Penman, 1966), indicating that this is a conserved feature of eukaryotic ribosome synthesis.

Previous proteomic analyses indicated that the released 90S preribosomes, which are presumably the substrates for RTC, contain many 40S synthesis factors but largely lack 60S processing factors (Grandi et al., 2002). This indicates substantial differences in the timing of association of assembly factors with the posttranscriptionally processed pre-rRNA population. Pre-60S particles can be isolated that still contain snoRNPs (Dez et al., 2004), consistent with the delayed modification of a fraction of the population. However, the data presented here reveal that most snoRNPs must associate with the nascent pre-rRNA transcripts. This has important consequences for our understanding of the entire pre-rRNA folding and assembly pathway.

## Experimental Procedures

### Yeast Strains

Analyses were performed in strain YMK120 (W303; *MATa*, tTA in *LYS2* gene, *tetR'::URA3-KL* in *ura3*), which carries the tetracycline activator (tTA) and reverse tetracycline repressor (tetR′) (Bellí et al., 1998) integrated into the genome to allow future analyses of ribosome synthesis mutants.

### Pulse-Labeling Experiments

The cells were grown at temperatures indicated in a synthetic dropout media without uracil (Formedia). For pulse experiments, [5,6-^3^H]-uracil (Amersham) (1 mCi/25ml culture) was added to the exponentially growing cells (OD_600_ = 0.4). At given time points, 1 ml of the labeling culture was directly dispensed into 10 ml of ethanol prechilled on dry ice (in 15 ml tubes). The samples were transferred to room temperature until all frozen media had melted (∼5 min) and then spun at 3000 g for 5 min. Pellets were resuspended in 1 ml of ice-cold water (to remove precipitated ammonium sulfate from media) and transferred to 1.5 ml tubes and spun again. Total RNA was extracted from pelleted cells using zirconia beads as described previously (Tollervey, 1987). The obtained RNA was dissolved in 15 μl of water and 1 μl was loaded on 1.2% agarose or 8% polyacrylamide/urea gels and separated by size as previously described (Sambrook et al., 1989). The separated RNA was transferred to a nylon membrane using wet electrotransfer, and membrane was dried and exposed to imaging plates (Fuji).

### Quantification

Signal intensities were quantified using Fuji FLA-5100 scanner and AIDA software (Raytest). We used 1D quantification analysis. As can be seen from the gel figures, pulse labeling leads to a significant “background” corresponding to all labeled nascent rRNAs and other RNAs, especially at later time points. To properly correct the quantified signal for this “background,” a line around the bands of interest was drawn and quantified. Then the same lane was duplicated and juxtaposed directly above or below the quantified bands (where space allowed). The signal from this lane (a local background) was then subtracted from the signal obtained from bands corresponding to various rRNAs. In addition, mature 18S and 25S rRNA were further corrected for quenching as described in the text. In the methionine labeling experiments, intensity values obtained for different pre-rRNAs were divided by the number of known methylated residues (65 for 35S, 44 for 27S and 25S, and 21 for 20S and 18S pre-rRNAs).

### Fitting of the Model to the Data

The model was fitted to the data manually starting from values determined by previous estimates of pre-rRNA lifetimes. Visual inspection of the curves was used to evaluate the best fit for all intermediates with a unique set of parameters. The parameters were changed one by one in order to adjust the fit of curves. The minimum amount by which the parameters were changed was 5 s, as the experimental error most likely exceeds this value. The Excel Solver confirms that the visually found best fit is at a local minimum. Using the Excel Solver to find the best fit without constraints based on prior knowledge frequently resulted in the software getting “stuck” in various local minima, predicting values of parameters that were inconstant with other biological data. However, we cannot formally exclude the possibility that another solution of the model exists that might be biologically relevant.

## Figures and Tables

**Table 1:** Modeled Lifetimes and Processing Times for Pre-rRNA Intermediates Resolved by Metabolic Labeling

Pre-rRNA Species	Life/Processing Times (s)
35S	10
27SA-NTC	15
27SA-RTC	95
27SB	45
27SA - > 27SB	20
27SB - > 25S	10
20S	115
35S transcription time	170
Elongation rate	40 nt s^−1^
Total time for 25S via RTC	365
Total time for 25S via NTC	260
Total time for 18S via RTC	295
Total time for 18S via NTC	215

## Supplemental Information

Document S1. Two Figures and One Table

Document S2. Excel Labeling Model
