# Supplementary material for: Yeast Pre-rRNA Processing and Modification Occur Cotranscriptionally
Source: Mol Cell. 2010 Mar 26;37(6):809–20. doi: 10.1016/j.molcel.2010.02.024 (PMC2860240; doi:10.1016/j.molcel.2010.02.024)
Supplement: Document S1. Two Figures and One Table [file mmc1.pdf]

Molecular Cell, Volume *37*

Supplemental Information

Yeast Pre-rRNA Processing and Modification Occur Cotranscriptionally

Martin Koš and David Tollervey

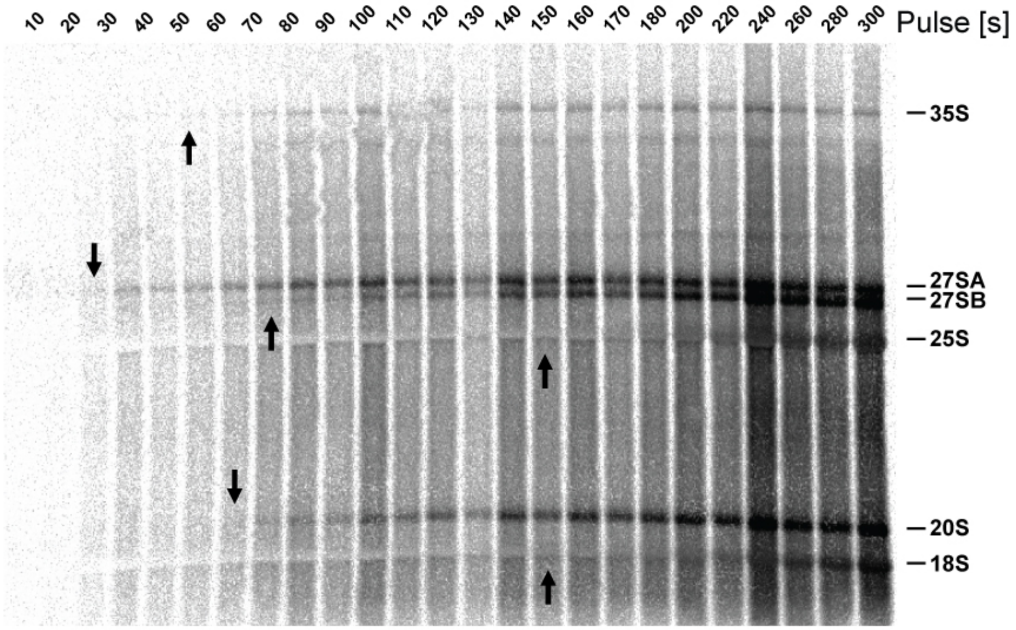

Figure S1 (related to Fig. 1). Metabolic labeling with [^{3}H]-adenine.

Cells were labeled with [^{3}H]-adenine and harvested by fast sampling technique. Total RNA was separated by electrophoresis in an agarose gel. Times of first detection of individual pre-rRNA processing intermediates are indicated by arrows.

Supplementary Figure S1
Adenine labelling

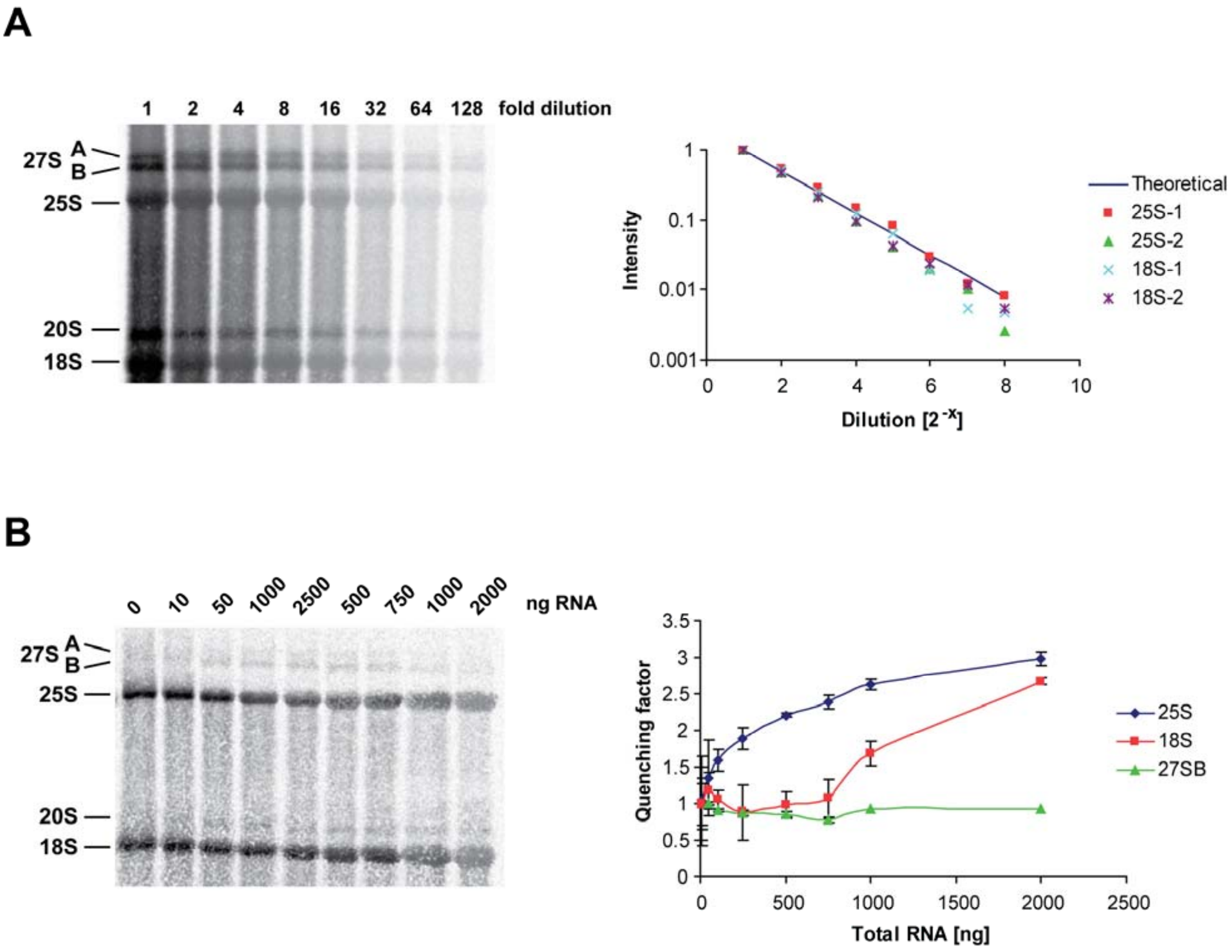

Figure S2 (related to Fig. 3). Quenching of ^{3}H signals by 25S and 18S rRNAs.

A: Two-fold dilution series of [^{3}H]-labeled RNA. Total RNA was extracted from cells labeled for 5 minutes with [^{3}H]-uracil. This RNA was diluted by total RNA extracted from non-labeled cells and 2 µg of total RNA were loaded per each lane. Here the fraction of [^{3}H]-labeled RNA is changing, whereas the total amount of RNA is constant. B: A constant amount of [^{3}H]-labeled RNA (5 ng) was mixed with increasing amounts of cold total RNA. The quenching factor was calculated as the ratio between the signal intensity in the first lane (only [^{3}H]-labeled RNA) and other lanes (mixed with cold RNA). While the signal for 27SB pre-rRNA was not affected by increasing amounts of cold RNA, the 25S and 18S rRNAs were significantly quenched.

Figure S2

Table S1. Mathematical model for pre-rRNA processing.

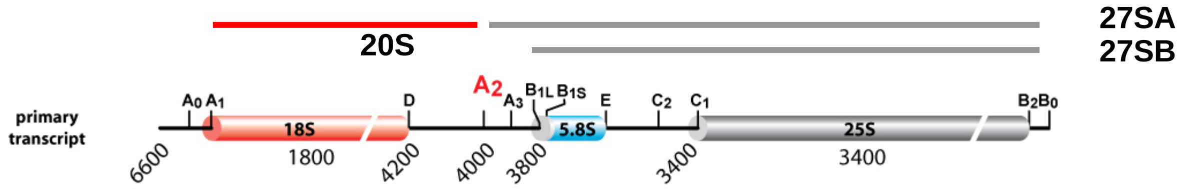

1) $I_{35S} = \sum_{t-\tau 35}^{t} I_{35S}^{t} - l$

- $0 < vt < 6600 \Rightarrow I_{35S}^{t} = vt \times (1 - \mathrm{P})$
- $vt \geq 6600 \Rightarrow I_{35S}^{t} = 6600 \times (1 - \mathrm{P})$

2) $I_{27SA} = \sum_{t-\tau 27SAa}^{t} I_{27SA\_RTC}^{t} + \sum_{t-\tau 27SAb}^{t} I_{27SA\_NTC}^{t} - l$

- $0 < vt < 4000 \Rightarrow I_{27SA\_RTC}^{t} = v(t - \tau 35S) \times (1 \text{-} \mathrm{P})$ and $I_{27SA\_NTC}^{t} = vt \times P$
- $vt > 4000 \Rightarrow I_{27SA\_NTC}^{t} = 4000 \times (1 - P)$ and for
  $v(t - \tau 35S) > 4000 \Rightarrow I_{27SA\_RTC}^{t} = 4000 \times P$

3) $I_{27SB} = \sum_{t-\tau 27SB}^{t} I_{27SB\_RTC}^{t} + \sum_{t-\tau 27SB}^{t} I_{27SB\_NTC}^{t} - l$

- $0 < v(t - \tau 35S - \tau 27SA_a - T_{27SA \to 27SB}) < 3800 \Rightarrow I_{27SB\_RTC}^{t} = v(t - \tau 35S - \tau 27SA - T_{27SA \to 27SB}) \times (1 - P)$
- $0 < v(t - \tau 27SA_b - T_{27SA \to 27SB}) < 3800 \Rightarrow I_{27SB\_NTC}^{t} = v(t - \tau 27SA_b - T_{27SA \to 27SB}) \times P$
- Otherwise $I_{27SB\_RTC}^{t} = 3800 \times (1 - P)$ and $I_{27SB\_NTC}^{t} = 3800 \times P$ respectively

4) $I_{25S} = \sum_{t-\tau 25S}^{t} I_{25S\_RTC}^{t} + \sum_{t-\tau 25S}^{t} I_{25S\_NTC}^{t} - l$

- $v(t - \tau 35S - \tau 27SA_a - \tau 27SB - T_{27SB \to 25S}) < 3400 \Rightarrow I_{25S\_RTC}^{t} = v(t - \tau 35S - \tau 27SA_a - \tau 27SB - T_{27SB \to 25S}) \times (1 - P)$
- $v(t - \tau 27SA_b - \tau 27SB - T_{27SB \to 25S}) < 3400 \Rightarrow I_{25S\_NTC}^{t} = v(t - \tau 27SA_b - \tau 27SB - T_{27SB \to 25S}) \times P$

- Otherwise $I^t_{25S\_NTC} = 3400 \times (1-P)$ and $I^t_{25S\_NTC} = 3400 \times P$ respectively

5) $I_{20S} = \sum_{t-\tau_{20S}}^{t} I^t_{20S\_RTC} + \sum_{t-\tau_{20S}}^{t} I^t_{20S\_NTC} - l$

- For $0 < v(t-\tau_{35S}) < 4000$ and
  - o $0 < v(t-\frac{L}{v}) < 2000 \Rightarrow I^t_{20S\_RTC} = 0$ and $I^t_{20S\_NTC} = v(t-\frac{L}{v}) \times \mathrm{P}$
  - o $v(t-\frac{L}{v}) \geq 2000 \Rightarrow I^t_{20S\_RTC} = 0$ and $I^t_{20S\_NTC} = 2000 \times \mathrm{P}$
- $4000 < v(t-\tau_{35S}) < 6000 \Rightarrow I^t_{20S\_RTC} = [v(t-\tau_{35S}) - 4000] \times (1-\mathrm{P})$ and $I^t_{20S\_NTC} = 2000 \times \mathrm{P}$
- $v(t-\tau_{35S}) > 6000 \Rightarrow I^t_{20S\_RTC} = 2000 \times (1-P)$ and $I^t_{20S\_NTC} = 2000 \times \mathrm{P}$

6) $I_{18S} = \sum_{t-\tau_{18S}}^{t} I^t_{18S\_RTC} + \sum_{t-\tau_{18S}}^{t} I^t_{18S\_NTC} - l$

- For $0 < v(t-\tau_{35S}-\tau_{20S}) < 4200$ and
  - o $0 < v(t-\frac{L}{v}-\tau_{20S}) < 1800 \Rightarrow I^t_{18S\_RTC} = 0$ and $I^t_{18S\_NTC} = v(t-\frac{L}{v}-\tau_{20S}) \times \mathrm{P}$
  - o $v(t-\frac{L}{v}-\tau_{20S}) \geq 1800 \Rightarrow I^t_{18S\_RTC} = 0$ and $I^t_{18S\_NTC} = 1800 \times \mathrm{P}$
- $4200 < v(t-\tau_{35S}-\tau_{20S}) < 6000 \Rightarrow I^t_{18S\_RTC} = [v(t-\tau_{35S}-\tau_{20S}) - 4200] \times (1-\mathrm{P})$ and $I^t_{18S\_NTC} = 1800 \times \mathrm{P}$
- $v(t-\tau_{35S}-\tau_{20S}) > 6000 \Rightarrow I^t_{18S\_RTC} = 1800 \times (1-P)$ and $I^t_{18S\_NTC} = 1800 \times \mathrm{P}$

On the top the primary transcript (35S) with known cleavage and processing points. Corresponding processing intermediates are indicated above. Numbers below represent distance in nucleotides from the 3’ end.

Equations used in the pre-rRNA labeling and processing model are shown with evaluation conditions.
